# Organ-specific autoantibodies and autoimmune diseases in juvenile systemic lupus erythematosus and juvenile dermatomyositis patients

**DOI:** 10.1186/1546-0096-10-S1-A14

**Published:** 2012-07-13

**Authors:** Nadia E Aikawa, Adriana A Jesus, Bernadete L Liphaus, Bernadete L Liphaus, Clovis A Silva, Magda C Sampaio, Adriana M Sallum

**Affiliations:** 1 University of São Paulo, Sao Paulo, Brazil

## Purpose

The purpose of this study was to evaluate organ-specific autoantibodies and autoimmune diseases in JSLE and JDM patients.

## Methods

Forty-one JSLE and 41 JDM patients were investigated for serum autoantibodies associated with autoimmune hepatitis, primary biliary cirrhosis, type 1 diabetes mellitus (T1DM), autoimmune thyroiditis, autoimmune gastritis and celiac disease. Patients with positive organ-specific antibodies were assessed for the presence of the respective organ-specific autoimmune diseases.

## Results

Mean age at diagnosis was significantly higher in JSLE compared to JDM patients (10.3±3.4 vs. 7.3±3.1years, p=0.0001), whereas the mean disease duration was similar in both groups. The frequencies of organ-specific autoantibodies were similar between JSLE and JDM (p>0.05). Of note, a high prevalence of autoantibodies related to DM1 and autoimmune thyroiditis was observed in both groups (20% vs. 15%, p=0.77 and 24% vs. 15%, p=0.41; respectively). Higher frequencies of antinuclear antibody - ANA (93% vs. 59%, p=0.0006), anti-dsDNA (61% vs. 2%, p<0.0001), anti-Ro (35% vs. 0%, p<0.0001), anti-Sm (p=0.01), anti-RNP (p=0.02), anti-La (p=0.03) and IgG aCL (p=0.001) were observed in JSLE compared to JDM patients. Organ-specific autoimmune diseases were evidenced only in JSLE patients (24% vs. 0%, p=0.13). Two JSLE patients had T1DM associated with Hashimoto thyroiditis and another had subclinical thyroiditis. Other JSLE patient had celiac disease diagnosis based on iron deficiency anaemia, presence of anti-endomysial antibody, duodenal biopsy compatible to celiac disease and response to a gluten-free diet.

## Conclusion

Organ-specific diseases were observed solely in JSLE patients and required specific therapy. The presence of these antibodies recommends the evaluation of organ-specific diseases and a rigorous follow-up of these patients.

## Disclosure

Nadia E. Aikawa: None; Adriana A. Jesus: None; Bernadete L. Liphaus: None; Bernadete L. Liphaus: None; Clovis A. Silva: None; Magda C. Sampaio: None; Adriana M. Sallum: None.

